# Plasma Monocyte Chemoattractant Protein-1 Level as a Predictor of the Severity of Community-Acquired Pneumonia

**DOI:** 10.3390/ijms17020179

**Published:** 2016-01-29

**Authors:** Kok-Khun Yong, Jer-Hwa Chang, Ming-Hsien Chien, Shih-Ming Tsao, Ming-Chih Yu, Kuan-Jen Bai, Thomas Chang-Yao Tsao, Shun-Fa Yang

**Affiliations:** 1 Institute of Medicine, Chung Shan Medical University, Taichung 402, Taiwan; khun1110@ms42.hinet.net; 2 Division of Pulmonary Medicine, Puli Christian Hospital, Puli Township, Nantou 54546, Taiwan; 3 Division of Pulmonary Medicine, Department of Internal Medicine, Wan Fang Hospital, Taipei Medical University, Taipei 116, Taiwan; m102094030@tmu.edu.tw (J.-H.C.); yutbc@tmu.edu.tw (M.-C.Y.); bkj@tmu.edu.tw (K.-J.B.); 4 School of Respiratory Therapy, College of Medicine, Taipei Medical University, Taipei 110, Taiwan; 5 Graduate Institute of Clinical Medicine, Taipei Medical University, Taipei 110, Taiwan; mhchien1976@gmail.com; 6 Department of Medical Education and Research, Wan Fang Hospital, Taipei Medical University, Taipei 116, Taiwan; 7 Institute of Biochemistry, Microbiology and Immunology, Chung Shan Medical University, Taichung 402, Taiwan; tsmhwy@ms24.hinet.net; 8 Division of Chest, Department of Internal Medicine, Chung Shan Medical University Hospital, Taichung 402, Taiwan; 9 Department of Medical Research, Chung Shan Medical University Hospital, Taichung 402, Taiwan

**Keywords:** community-acquired pneumonia, pneumonia severity index, monocyte chemoattractant protein-1, biochemical marker

## Abstract

Monocyte chemoattractant protein (MCP)-1 increases in the serum of immunocompetent patients with community-acquired pneumonia (CAP). However, the correlation between the circulating level of MCP-1 and severity of CAP remains unclear. This study investigated differential changes in the plasma MCP-1 levels of patients with CAP before and after an antibiotic treatment and further analyzes the association between the CAP severity and MCP-1 levels. We measured the plasma MCP-1 levels of 137 patients with CAP and 74 healthy controls by using a commercial enzyme-linked immunosorbent assay. Upon initial hospitalization, Acute Physiology and Chronic Health Evaluation II (APACHE II); confusion, urea level, respiratory rate, blood pressure, and age of >64 years (CURB-65); and pneumonia severity index (PSI) scores were determined for assessing the CAP severity in these patients. The antibiotic treatment reduced the number of white blood cells (WBCs) and neutrophils as well as the level of C-reactive protein (CRP) and MCP-1. The plasma MCP-1 level, but not the CRP level or WBC count, correlated with the CAP severity according to the PSI (*r* = 0.509, *p* < 0.001), CURB-65 (*r* = 0.468, *p* < 0.001), and APACHE II (*r* = 0.360, *p* < 0.001) scores. We concluded that MCP-1 levels act in the development of CAP and are involved in the severity of CAP.

## 1. Introduction

Community-acquired pneumonia (CAP) is caused by exposure to bacterial agents outside of a healthcare setting. CAP is the first reason of death from infectious diseases globally and causes a great burden on health care resources [1]. In the United States, CAP along with influenza ranks the eighth among the causes of death [2]. In Taiwan, pneumonia was the fourth leading cause of death in 2014, according to the statistics of the Ministry of Health and Welfare [3]. Thus, assessing the severity of the disease and predicting the outcome are necessary for adequately allocating health care resources and treatment options for managing CAP. At present, the white blood cell (WBC) count and C-reactive protein (CRP) level are the commonly-used laboratory values for diagnosing CAP and following up on patients with CAP [4]. However, the specificity and sensitivity of these diagnostic markers are inadequate, particularly for predicting CAP severity [5,6]. In addition, the confusion, urea level, respiratory rate, blood pressure, and age of >64 years (CURB-65) scores and the pneumonia severity index (PSI) are the most CAP-specific scoring systems for predicting CAP outcomes; however, these systems are subjective and have some limitations [7]. For instance, PSI requires a two-step algorithm, integrating clinical signs, and demographic and laboratory data for the assessment [8]. PSI is highly complex, disadvantaging its dissemination and execution, particularly in routine clinical practice. The disadvantages of both CURB-65 and PSI scores include the usage of the most routine clinical and laboratory data, which require assessments of patients with CAP without delay. Moreover, similar clinical signs are manifested by other diseases, such as acute bronchitis, an acute exacerbation of chronic obstructive pulmonary disease, and congestive heart failure [9]. Therefore, searching for novel biological markers for an early and rapid diagnosis of CAP and for detecting the severity of CAP is required.

Monocyte chemoattractant protein (MCP)-1, also known as chemokine C–C motif ligand-2, is produced by various cells, such as monocytes, macrophages, lymphocytes, and airway epithelial cells in response to inflammation [10,11,12]. MCP-1 has been demonstrated to be a potent monocyte and macrophage [13], neutrophil [14], and T-cell [15] chemoattractant against bacterial pulmonary infection; this effect is due to MCP-1 binding to its sole receptor chemokine C–C motif receptor 2 (CCR2) [16]. For instance, CCR2-deficient mice exhibited impairment in macrophage migration, as well as the clearance of bacteria, from the pulmonary and extrapulmonary organs after an intravenous challenge with *Listeria monocytogenes* [17]. Previous studies have indicated that neutrophils express CCR2 [18] and demonstrate chemotaxis toward MCP-1, which can act against acute bacterial pneumonia [14]. The host defense mechanism against bacterial lung infections depends on effective recruitment of neutrophils, monocytes, and macrophages at the site of infection [19]. On the basis of its characteristics, we hypothesized that MCP-1 is a biochemical marker for an early diagnosis of CAP and for detecting the severity of CAP.

Previous studies have indicated that the levels of MCP-1 in blood are related with the severity of coronary artery disease in patients with chronic kidney disease [20] and with the disease activity of rheumatoid arthritis [21]. MCP-1 is known as a prognostic factor for lung, cervical, and ovarian cancers [22,23,24]. In addition, MCP-1 levels in the bronchoalveolar lavage fluid were shown to correlate with interstitial lung disease severity [25]. However, to our knowledge, no cohort study has reported the prognostic benefit of MCP-1 in patients with CAP. Therefore, we measured the plasma MCP-1 levels in a CAP cohort and in healthy controls for evaluating whether MCP-1 could be a beneficial biochemical marker to aid differentiation between controls and patients with CAP, as well as clarify any association between the circulating MCP-1 levels and CAP severity.

## 2. Results

Demographic data and clinical characteristics of the participants are presented in Table 1. In total, 211 participants were included in the analysis, and age and sex were not significantly different between patients with CAP and the controls (Table 1). Among the 137 patients with CAP, the mean PSI, CURB-65, and APACHE II scores were 83.89 ± 36.63, 1.12 ± 0.94, and 10.20 ± 5.27, respectively. Moreover, patients with CAP exhibited significantly higher CRP levels (median, 10.10 *vs.* 0.40 mg/dL, *p* < 0.001), WBC counts (median, 12,030 *vs.* 5900 cells/mm^3^, *p* < 0.001), and neutrophil counts (median, 8715 *vs.* 3580 cells/mm^3^, *p* < 0.001) than did the controls (Table 1). In these patients, the antibiotic treatment significantly reduced the CRP levels (before antibiotic treatment: median, 10.10 mg/dL and after antibiotic treatment: median, 2.10 mg/dL; *p* < 0.001), WBC counts (before antibiotic treatment: median, 12,030 cells/mm^3^ and after antibiotic treatment: median 8280 cells/mm^3^; *p* < 0.001), and neutrophil counts (before antibiotic treatment: median, 8715 cells/mm^3^ and after antibiotic treatment: median, 5616 cells/mm^3^; *p* < 0.001) (Table 1).

**Table 1:** Laboratory data of both controls and patients with community-acquired pneumonia (CAP) before and after they received treatment ^a^.

Clinical Variable	Controls (*n* = 74) Median (Range)	Before Antibiotic Treatment (*n* = 137) Median (Range)	After Antibiotic Treatment (*n* = 137) Median (Range)	*p* Value UT/C ^b^	*p* Value UT/T ^c^
Age	59.89 ± 11.04 ^d^	64.89 ± 20.89 ^d^	-	*p* = 0.057	-
Gender	-	-	-		-
Male	49 (66.2%)	87 (63.5%)	-	*p* = 0.694	-
Female	25 (33.8%)	50 (36.5%)	-	-	-
CRP (mg/dL)	0.40 (0.2–1.50)	10.1 (0.50–33.30)	2.10 (0.10–17.20)	*p* < 0.001	*p* < 0.001
WBCs (cells/mm^3^)	5900 (2980–13,700)	12,030 (3560–32,480)	8280 (3300–28,180)	*p* < 0.001	*p* < 0.001
Neutrophils (cells/mm^3^)	3580 (1078–9946)	8715 (1032–29,686)	5616 (1518–25,841)	*p* < 0.001	*p* < 0.001
PSI score	-	83.89 ± 36.63 ^d^	-	-	-
CURB-65 score	-	1.12 ± 0.94 ^d^	-	-	-
APACHE II score	-	10.20 ± 5.27 ^d^	-	-	-
Hospital length of stay (Days)	-	10.37 ±14.54 ^d^	-	-	-

CRP, C-reactive protein; WBCs, white blood cells; C, controls; UT, patients with CAP before they received antibiotic treatment; T, patients with CAP after they received antibiotic treatment; ^a^ *p* < 0.05 was considered significant; ^b^ The statistical difference was analyzed by the Mann–Whitney *U*-test; ^c^ The statistical difference was analyzed by the Wilcoxon signed-ranks test; ^d^ Mean ± S.D.

**Figure 1:**
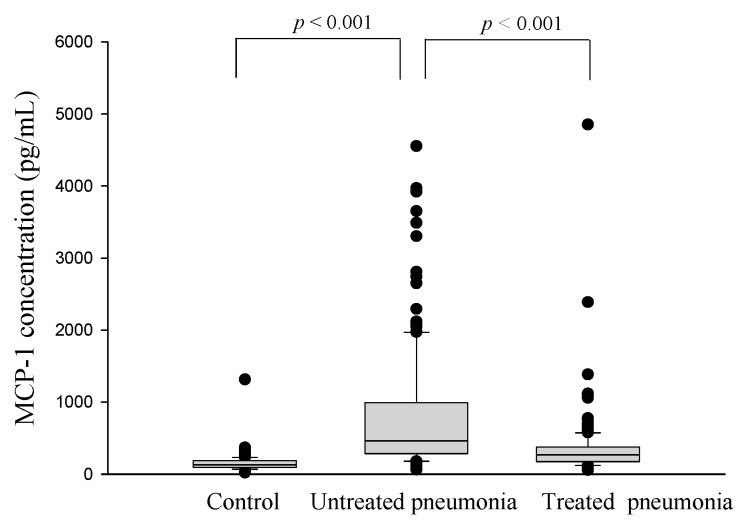
Levels of plasma monocyte chemoattractant protein (MCP)-1 in healthy controls and in patients with community-acquired pneumonia (CAP) before and after antibiotic treatment. The plasma MCP-1 level was significantly elevated in patients with CAP before they received treatment compared with the controls (*p* < 0.001) and significantly decreased in patients with CAP after treatment (*p* < 0.001).

Figure 1 presents the MCP-1 levels in patients with CAP and the controls. The patients exhibited significantly higher plasma MCP-1 levels than did the controls (controls: 163.3 ± 17.7 pg/mL and patients: 803.2 ± 74.1 pg/mL; *p* < 0.001; Figure 1). In patients with CAP, the antibiotic treatment significantly reduced the expression of MCP-1 (before antibiotic treatment: 803.2 ± 74.1 pg/mL and after antibiotic treatment: 359.4 ± 40.5 pg/mL; *p* < 0.001; Figure 1).

**Figure 2:**
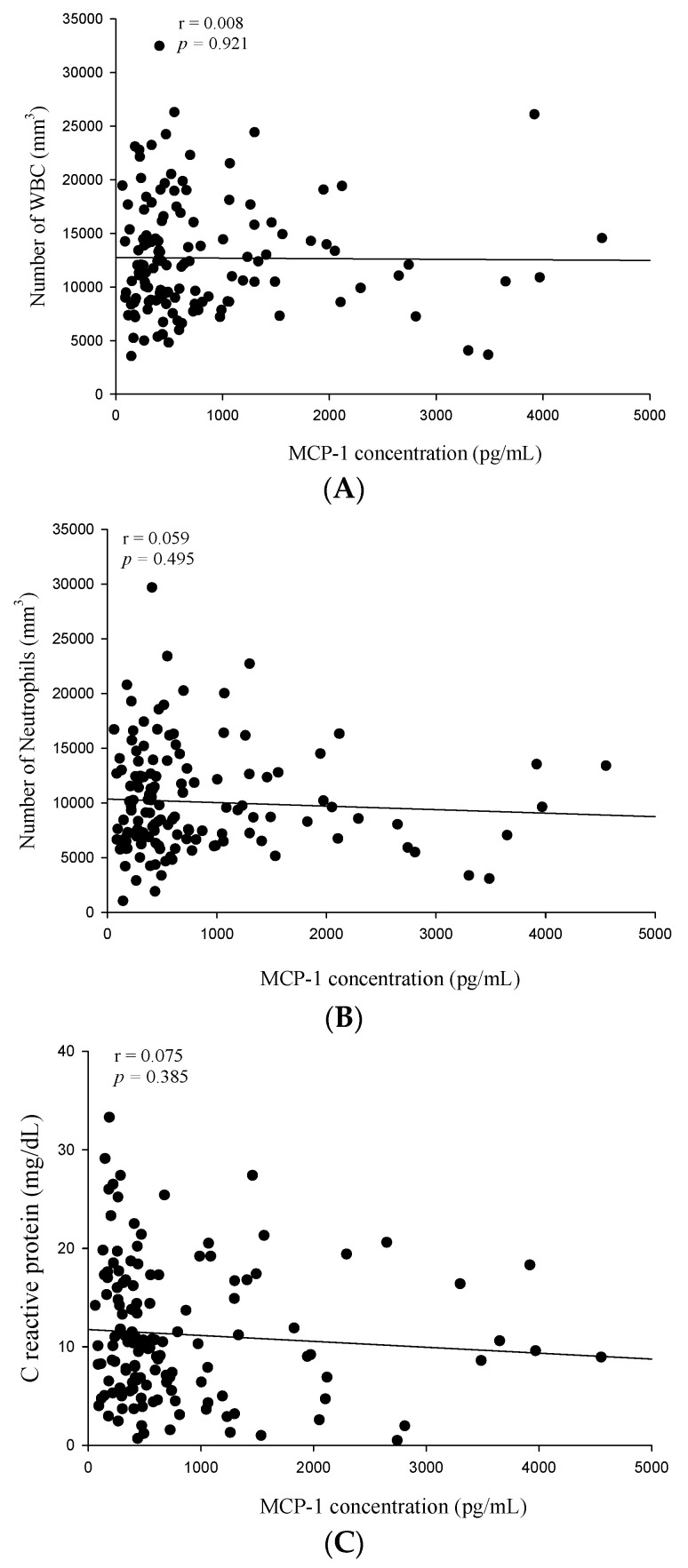
Correlations of plasma monocyte chemoattractant protein (MCP)-1 with white blood cells (WBCs), neutrophils, and C-reactive protein (CRP) in 137 patients with community-acquired pneumonia (CAP). (**A**) No significant correlation was observed between the pretreatment plasma MCP-1 levels and WBC counts (Spearman’s correlation coefficients: *r* = 0.008, *p* = 0.921, *n* = 137); (**B**) no significant correlation was observed between the pretreatment plasma MCP-1 levels and neutrophil counts (Spearman’s correlation coefficients: *r* = 0.059, *p* = 0.495, *n* = 137); and (**C**) no significant correlation was observed between the pretreatment plasma MCP-1 levels and CRP levels (Spearman’s correlation coefficients: *r* = 0.075, *p* = 0.385, *n* = 137).

WBC and neutrophil counts as well as CRP and MCP-1 levels were elevated in patients with CAP before the antibiotic treatment. Therefore, we further analyzed the correlation between MCP-1 levels and WBC counts, neutrophil counts, and CRP levels in patients with CAP. No significant correlation was observed between MCP-1 levels and WBC counts (Spearman’s correlation coefficient: *r* = 0.008, *p* = 0.921; Figure 2A), neutrophil counts (Spearman’s correlation coefficient: *r* = 0.059, *p* = 0.495; Figure 2B), or CRP levels (Spearman’s correlation coefficient: *r* = 0.075, *p* = 0.385; Figure 2C) before the antibiotic treatment in patients with CAP.

**Figure 3:**
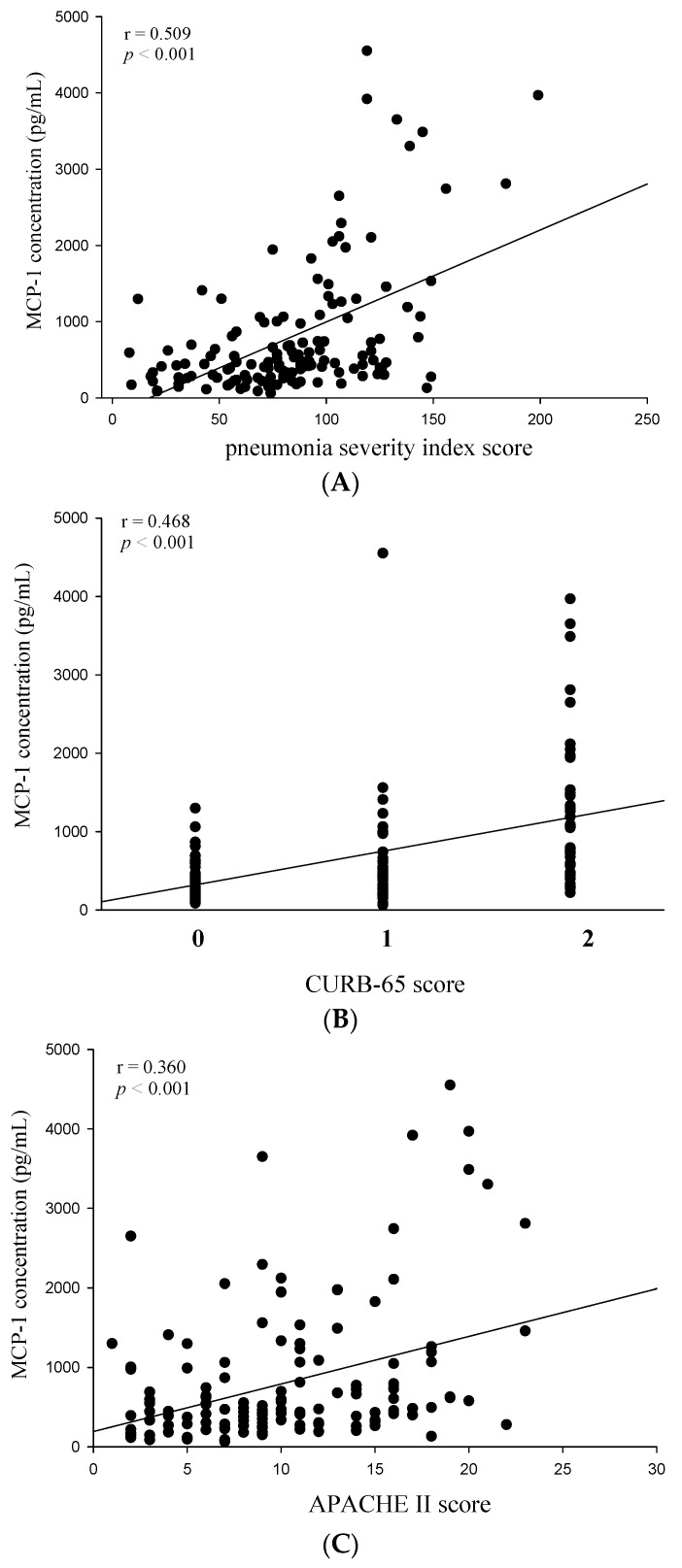
Correlations of plasma monocyte chemoattractant protein (MCP)-1 with the pneumonia severity index (PSI), confusion, urea level, respiratory rate, blood pressure, and age of >64 years (CURB-65), and Acute Physiology and Chronic Health Evaluation II (APACHE II) scores in 137 patients with community-acquired pneumonia (CAP). (**A**) A significantly positive correlation was observed between plasma MCP-1 levels and PSI scores (Spearman’s correlation coefficients: *r* = 0.509, *p* < 0.001); (**B**) A significantly positive correlation was observed between plasma MCP-1 levels and CURB-65 scores (Spearman’s correlation coefficients: *r* = 0.468, *p* < 0.001); (**C**) A positive correlation was observed between plasma MCP-1 levels and APACHE II scores (Spearman’s correlation coefficients: *r* = 0.360, *p* < 0.001).

Next, we investigated the correlation between the MCP-1 and CRP circulating levels and WBC counts and the CAP severity in patients before the antibiotic treatment by using the PSI, CURB-65, and APACHE II scoring systems. Correlations among PSI, CURB-65, and APACHE II scores and MCP-1 levels in patients with CAP before receiving the antibiotic treatment are presented in Figure 3. Significant correlations were observed between MCP-1 levels and PSI scores (Spearman’s correlation coefficient: *r* = 0.509, *p* < 0.001; Figure 3A), CURB-65 scores (Spearman’s correlation coefficient: *r* = 0.468, *p* < 0.001; Figure 3B), and APACHE II scores (Spearman’s correlation coefficient: *r* = 0.360, *p* < 0.001; Figure 3C).

In contrast to MCP-1, neither CRP levels nor WBC counts correlated with the CAP severity in patients, as evidenced from the statistically non-significant correlations among PSI, CURB-65, and APACHE II scores and CRP levels (*p* = 0.926, 0.824, and 0.968, respectively) and WBC counts (*p* = 0.973, 0.544, and 0.416, respectively; Table 2). In addition, MCP-1 and CRP levels and WBC counts did not correlate significantly with the length of hospital stay (Table 2).

**Table 2:** Correlation of white blood cells (WBCs), C-reactive protein (CRP), and monocyte chemoattractant protein-1 (MCP-1) with clinical pathological features.

Variable	WBC (*n* = 137)		CRP (*n* = 137)		MCP-1 (*n* = 137)	
	*r*	*p* Value	*r*	*p* Value	*r*	*p* Value
PSI score	−0.034	0.690	−0.007	0.931	0.509	<0.001
CURB-65 score	0.036	0.674	0.027	0.758	0.468	<0.001
APACHE II score	0.050	0.563	0.015	0.859	0.360	<0.001
Length of hospital stay	−0.040	0.641	0.024	0.779	0.049	0.567

PSI, Pneumonia Severity Index; APACHE II, Acute Physiology and Chronic Health Evaluation II.

## 3. Discussion

Clinical guidelines for managing adult patients with CAP suggest using a severity-based approach for guiding therapeutic options, such as the need for hospital or ICU admission, suitability for ambulatory care, and choice and route of antimicrobial agents. The pretreatment CRP levels and WBC and neutrophil counts were significantly higher in patients with CAP before treatment than in the controls; the antibiotic treatment significantly reduced those parameters in the same patients. In accordance with our findings, previous studies have reported that CRP levels may contribute to establishing a CAP diagnosis [26,27]. However, inconsistent results have been reported by studies evaluating the use of CRP as a prognostic factor for CAP. For instance, some studies have indicated that CRP is a promising diagnostic and prognostic tool for managing CAP [28,29]. However, other studies have indicated that elevated CRP levels in patients with CAP have no prognostic relevance [30,31]. For example, Brunkhorst *et al.*, found that CRP concentration was not significantly associated with a change in the clinical classification or death of the patients with severe pneumonia [30]. Thiem *et al.*, reported that no association was found between CRP or WBC and mortality in elderly hospitalized patients with CAP [31]. For instance, CRP levels are typically high in respiratory tract infections, even in patients exhibiting moderately severe CAP [32]. In patients with pneumococcal CAP, despite their having high levels of bacterial antigens, relatively weak CRP responses are observed in the majority of severe cases [33].

In patients with CAP in this study, no significant correlation was observed between the CRP levels or WBC counts and CAP severity indices (PSI, CURB-65, and APACHE II). In contrast to CRP, we observed that high MCP-1 values were associated with several variables (PSI, CURB-65, and APACHE II), thus indicating the disease severity. Moreover, we found higher plasma MCP-1 levels in patients with CAP than in healthy controls, and the antibiotic treatment significantly reduced these levels in the same patients. The lack of correlation between the CRP and MCP-1 values in this study indicates different pathophysiological mechanisms of the two parameters. CRP is induced by interleukin (IL)-6, IL-1β, and tumor necrosis factor-α [34]. Although MCP-1 can also be induced by these proinflammatory cytokines, CRP is almost exclusively synthesized by hepatocytes under stimulation by inflammatory cytokines [35]. However, MCP-1 is expressed by airway epithelial cells in response to inflammatory stimuli [11]. We suggest that MCP-1 acts as a more direct reflective marker than does CRP with respect to the response to an inflammatory stimulus in lung tissues and also as a more specific marker of the diagnosis and clinical assessment of CAP severity in Taiwanese populations. Moreover, although our current data shown that there is some significant positive correlation between MCP-1 expression and the PSI, CURB-65, and APACHE II scores in CAP patients. However, we found that MCP-1 concentration did not correlate significantly with the length of hospital stay. This discrepancy may have been due to the small sample size or racial differences.

Predicting the severity and identifying the etiology of CAP is crucial for managing CAP. Identifying the etiology of CAP is clinically difficult because single clinical, radiological, or laboratory parameters have limited value for predicting the infectious organism [36], and no rapid test has been standardized for diagnosing atypical or viral pathogens. Therefore, a type of empirical broad-spectrum antibiotic therapy is typically selected [37]. Although the CRP level and WBC count are used for diagnosing pulmonary infections, they are nonspecific and not helpful in differentiating bacterial and viral etiologies of pneumonia [38]. In addition to the traditional biomarkers, such as WBC and CRP, other biomarkers, such as procalcitonin (PCT), long pentraxin 3, and YKL-40 have been recently reported as potential tools for predicting CAP severity [9,39,40]. Among these biomarkers, only PCT was reported as a potential predictor of etiology in patients with CAP [41]. To date, whether MCP-1 can selectively recognize different types of pathogens remains unclear. However, a previous report indicated that MCP-1 demonstrated a positive correlation with PCT levels in patients with a community-acquired bacterial infection [42], suggesting that MCP-1 is also a predictor of etiology in patients with CAP. One of the limitations of this study is the lack of microbial data; different pathogens may have different impacts on the MCP-1 expression. Therefore, future studies are needed to distinguish the relationship between MCP-1 and different microbial pathogens that cause CAP. Another limitation of our study is the low sample size. Hence, the results should be confirmed in a larger population.

## 4. Materials and Methods

### 4.1. Participants and Diagnoses

This cohort study was conducted from January 2009 to December 2012 by the Department of Medical Research and Departments of Infectious Diseases and Chest Medicine, Chung Shan Medical University Hospital (CSMUH) Taichung, Taiwan. This study was approved by the Institutional Review Board (IRB) of CSMUH (IRB no. CS11237; 9 March 2012). The inclusion criteria comprised patients aged >20 years, who were diagnosed by the emergency room or outpatient department, and who were admitted for the management of CAP. Demographic characteristics, comorbidities, symptoms and signs of pneumonia, laboratory results, and previous antibiotic treatment were collected upon admission. Exclusion criteria included being an outpatient; having been transferred from another hospital; having had a separate hospital admission in the previous three weeks because of other acute conditions, such as pulmonary edema, pulmonary embolism, or a malignancy appearing during follow-up; pneumonia caused by tuberculosis or a malignancy; being severely immunocompromised, including severe neutropenia (WBC count, <10^9^ cells/L); and having an organ or bone marrow transplant, or HIV infection. All of the CAP patients were given antibiotics intravenously within first 48 h. Thereafter, oral antibiotics were given based on standard guidelines. The pretreatment blood samples were collected before the patients with CAP received treatment protocols, and post-treatment blood samples were obtained within three days when the pneumonia had been cured. The pneumonia severity was assessed using the PSI [8], Acute Physiology and Chronic Health Evaluation II (APACHE II) [43], and CURB-65 [44] scores.

### 4.2. Patients and Blood Sample Collection

We consecutively enrolled 137 patients with CAP and 74 healthy controls. People who visited the Department of Family and Community Medicine, CSMUH for a routine health examination were selected as the healthy controls. All patients with CAP were empirically treated with antimicrobial agents. We collected blood samples to determine the WBC and neutrophil counts as well as CRP and plasma MCP-1 levels in patients with CAP before and after an antibiotic treatment. Blood samples of the controls were also collected and examined. The blood samples were placed in tubes containing ethylenediaminetetraacetic acid, immediately centrifuged at 3000 rpm, and stored at −80 °C.

### 4.3. Measurements of the White Blood Cell (WBC) and Neutrophil Counts and C-Reactive Protein (CRP) Level

The WBC and neutrophil counts and CRP level were measured by the clinical laboratory staff members who were blinded to the source of the study samples.

### 4.4. Measurement of Plasma MCP-1 Levels

The MCP-1 levels in the plasma samples were analyzed using human MCP-1 enzyme-linked immunosorbent assay (ELISA) kits (R and D Systems, Abingdon, UK). The results were calculated from a standard curve generated by diluting a known amount of recombinant MCP-1 protein. Each standard or sample was assayed in duplicate.

### 4.5. Statistical Analyses

Statistical analyses were conducted using SPSS 15.0 statistical software (SPSS, Chicago, IL, USA). All continuous variables were expressed as the mean ± standard error, and the number (*n*) was expressed as percentages for categorical variables. To compare untreated patients and healthy controls, the Mann–Whitney *U* test was conducted for continuous variables that did not follow a parametric distribution, and the Wilcoxon signed-rank test was used for comparing the categorical variables of untreated and treated patients. A linear regression analysis was applied for assessing the correlations of MCP-1 with all the clinical and laboratory variables of patients with CAP. Statistical significance was defined as *p* < 0.05 in a two-tailed test.

## 5. Conclusions

In conclusion, in Taiwanese populations, plasma MCP-1 levels can be used for predicting CAP severity with higher sensitivity than that of CRP. Plasma MCP-1 can also be applied to differentiate patients with CAP from healthy participants and assess the effects of antibiotic treatment on patients with CAP. In this study, we revealed that detecting plasma MCP-1 levels can be useful in clinically managing CAP.

## Abbreviations

MCP-1: Monocyte chemoattractant protein-1; CAP: community-acquired pneumonia; PSI: Pneumonia Severity Index; APACHE II: Acute Physiology and Chronic Health Evaluation II; WBC: white blood cells; CRP: C-reactive protein.

## References

[1] Mandell L.A., Wunderink R.G., Anzueto A., Bartlett J.G., Campbell G.D., Dean N.C., Dowell S.F., File T.M., Musher D.M., Niederman M.S. (2007). Infectious diseases society of america/american thoracic society consensus guidelines on the management of community-acquired pneumonia in adults. Clin. Infect. Dis..

[2] Ramirez J.A., Anzueto A.R. (2011). Changing needs of community-acquired pneumonia. J. Antimicrob. Chemother..

[3] Department of Statistics of Ministry of Health and Welfare in Taiwan (2013). Causes of Death in Taiwan, 2012.

[4] Woodhead M., Blasi F., Ewig S., Garau J., Huchon G., Ieven M., Ortqvist A., Schaberg T., Torres A., van der Heijden G. (2011). Guidelines for the management of adult lower respiratory tract infections—Full version. Clin. Microbiol. Infect..

[5] Almirall J., Bolibar I., Toran P., Pera G., Boquet X., Balanzo X., Sauca G. (2004). Contribution of C-reactive protein to the diagnosis and assessment of severity of community-acquired pneumonia. Chest.

[6] Holm A., Nexoe J., Bistrup L.A., Pedersen S.S., Obel N., Nielsen L.P., Pedersen C. (2007). Aetiology and prediction of pneumonia in lower respiratory tract infection in primary care. Br. J. Gen. Pract..

[7] Huang D.T., Weissfeld L.A., Kellum J.A., Yealy D.M., Kong L., Martino M., Angus D.C., Gen I.M.S.I. (2008). Risk prediction with procalcitonin and clinical rules in community-acquired pneumonia. Ann. Emerg. Med..

[8] Fine M.J., Auble T.E., Yealy D.M., Hanusa B.H., Weissfeld L.A., Singer D.E., Coley C.M., Marrie T.J., Kapoor W.N. (1997). A prediction rule to identify low-risk patients with community-acquired pneumonia. N. Engl. J. Med..

[9] Muller B., Harbarth S., Stolz D., Bingisser R., Mueller C., Leuppi J., Nusbaumer C., Tamm M., Christ-Crain M. (2007). Diagnostic and prognostic accuracy of clinical and laboratory parameters in community-acquired pneumonia. BMC Infect. Dis..

[10] Standiford T.J., Kunkel S.L., Phan S.H., Rollins B.J., Strieter R.M. (1991). Alveolar macrophage-derived cytokines induce monocyte chemoattractant protein-1 expression from human pulmonary type II-like epithelial cells. J. Biol. Chem..

[11] Lundien M.C., Mohammed K.A., Nasreen N., Tepper R.S., Hardwick J.A., Sanders K.L., van Horn R.D., Antony V.B. (2002). Induction of MCP-1 expression in airway epithelial cells: Role of CCR2 receptor in airway epithelial injury. J. Clin. Immunol..

[12] Yoo J.K., Kwon H., Khil L.Y., Zhang L., Jun H.S., Yoon J.W. (2005). IL-18 induces monocyte chemotactic protein-1 production in macrophages through the phosphatidylinositol 3-kinase/Akt and MEK/ERK1/2 pathways. J. Immunol..

[13] Moore T.A., Standiford T.J. (2001). Cytokine immunotherapy during bacterial pneumonia: From benchtop to bedside. Semin. Respir. Infect..

[14] Balamayooran G., Batra S., Balamayooran T., Cai S., Jeyaseelan S. (2011). Monocyte chemoattractant protein 1 regulates pulmonary host defense via neutrophil recruitment during escherichia coli infection. Infect. Immun..

[15] Carr M.W., Roth S.J., Luther E., Rose S.S., Springer T.A. (1994). Monocyte chemoattractant protein 1 acts as a T-lymphocyte chemoattractant. Proc. Natl. Acad. Sci. USA.

[16] Murphy P.M., Baggiolini M., Charo I.F., Hebert C.A., Horuk R., Matsushima K., Miller L.H., Oppenheim J.J., Power C.A. (2000). International union of pharmacology. XXII. Nomenclature for chemokine receptors. Pharmacol. Rev..

[17] Kurihara T., Warr G., Loy J., Bravo R. (1997). Defects in macrophage recruitment and host defense in mice lacking the CCR2 chemokine receptor. J. Exp. Med..

[18] Iida S., Kohro T., Kodama T., Nagata S., Fukunaga R. (2005). Identification of CCR2, flotillin, and *gp49B* genes as new G-CSF targets during neutrophilic differentiation. J. Leukoc. Biol..

[19] Craig A., Mai J., Cai S., Jeyaseelan S. (2009). Neutrophil recruitment to the lungs during bacterial pneumonia. Infect. Immun..

[20] Akdogan M.F., Azak A., Denizli N., Huddam B., Kocak G., Gucun M., Tatlisu M.A., Demirci R., Yilmaz B., Dikec M. (2015). MCP-1 and soluble tweak levels are independently associated with coronary artery disease severity in patients with chronic kidney disease. Ren. Fail..

[21] Liou L.B., Tsai W.P., Chang C.J., Chao W.J., Chen M.H. (2013). Blood monocyte chemotactic protein-1 (MCP-1) and adapted disease activity score28-MCP-1: Favorable indicators for rheumatoid arthritis activity. PLoS ONE.

[22] Zhang X.W., Qin X., Qin C.Y., Yin Y.L., Chen Y., Zhu H.L. (2013). Expression of monocyte chemoattractant protein-1 and CC chemokine receptor 2 in non-small cell lung cancer and its significance. Cancer Immunol. Immunother..

[23] Lebrecht A., Hefler L., Tempfer C., Koelbl H. (2001). Serum cytokine concentrations in patients with cervical cancer: Interleukin-4, interferon-γ, and monocyte chemoattractant protein-1. Gynecol. Oncol..

[24] Hefler L., Tempfer C., Heinze G., Mayerhofer K., Breitenecker G., Leodolter S., Reinthaller A., Kainz C. (1999). Monocyte chemoattractant protein-1 serum levels in ovarian cancer patients. Br. J. Cancer.

[25] Hartl D., Griese M., Nicolai T., Zissel G., Prell C., Reinhardt D., Schendel D.J., Krauss-Etschmann S. (2005). A role for MCP-1/CCR2 in interstitial lung disease in children. Respir. Res..

[26] Smith R.P., Lipworth B.J. (1995). C-reactive protein in simple community-acquired pneumonia. Chest.

[27] Flanders S.A., Stein J., Shochat G., Sellers K., Holland M., Maselli J., Drew W.L., Reingold A.L., Gonzales R. (2004). Performance of a bedside C-reactive protein test in the diagnosis of community-acquired pneumonia in adults with acute cough. Am. J. Med..

[28] Ortqvist A., Hedlund J., Wretlind B., Carlstrom A., Kalin M. (1995). Diagnostic and prognostic value of interleukin-6 and C-reactive protein in community-acquired pneumonia. Scand. J. Infect. Dis..

[29] Hohenthal U., Hurme S., Helenius H., Heiro M., Meurman O., Nikoskelainen J., Kotilainen P. (2009). Utility of C-reactive protein in assessing the disease severity and complications of community-acquired pneumonia. Clin. Microbiol. Infect..

[30] Brunkhorst F.M., Al-Nawas B., Krummenauer F., Forycki Z.F., Shah P.M. (2002). Procalcitonin, C-reactive protein and apache II score for risk evaluation in patients with severe pneumonia. Clin. Microbiol. Infect..

[31] Thiem U., Niklaus D., Sehlhoff B., Stuckle C., Heppner H.J., Endres H.G., Pientka L. (2009). C-reactive protein, severity of pneumonia and mortality in elderly, hospitalised patients with community-acquired pneumonia. Age Ageing.

[32] Polzin A., Pletz M., Erbes R., Raffenberg M., Mauch H., Wagner S., Arndt G., Lode H. (2003). Procalcitonin as a diagnostic tool in lower respiratory tract infections and tuberculosis. Eur. Respir. J..

[33] Tateda K., Kusano E., Matsumoto T., Kimura K., Uchida K., Nakata K., Yamaguchi K. (2006). Semi-quantitative analysis of streptococcus pneumoniae urinary antigen: Kinetics of antigen titers and severity of diseases. Scand. J. Infect. Dis..

[34] Sheldon J., Riches P., Gooding R., Soni N., Hobbs J.R. (1993). C-reactive protein and its cytokine mediators in intensive-care patients. Clin. Chem..

[35] Harbarth S., Holeckova K., Froidevaux C., Pittet D., Ricou B., Grau G.E., Vadas L., Pugin J. (2001). Diagnostic value of procalcitonin, interleukin-6, and interleukin-8 in critically ill patients admitted with suspected sepsis. Am. J. Respir. Crit. Care Med..

[36] Simon L., Gauvin F., Amre D.K., Saint-Louis P., Lacroix J. (2004). Serum procalcitonin and C-reactive protein levels as markers of bacterial infection: A systematic review and meta-analysis. Clin. Infect. Dis..

[37] Whicher J., Bienvenu J., Monneret G. (2001). Procalcitonin as an acute phase marker. Ann. Clin. Biochem..

[38] Kruger S., Welte T. (2012). Biomarkers in community-acquired pneumonia. Expert Rev. Respir. Med..

[39] Kao S.J., Yang H.W., Tsao S.M., Cheng C.W., Bien M.Y., Yu M.C., Bai K.J., Yang S.F., Chien M.H. (2013). Plasma long pentraxin 3 (PTX3) concentration is a novel marker of disease activity in patients with community-acquired pneumonia. Clin. Chem. Lab. Med..

[40] Wang H.L., Hsiao P.C., Tsai H.T., Yeh C.B., Yang S.F. (2013). Usefulness of plasma YKL-40 in management of community-acquired pneumonia severity in patients. Int. J. Mol. Sci..

[41] Horie M., Ugajin M., Suzuki M., Noguchi S., Tanaka W., Yoshihara H., Kawakami M., Kichikawa Y., Sakamoto Y. (2012). Diagnostic and prognostic value of procalcitonin in community-acquired pneumonia. Am. J. Med. Sci..

[42] Holub M., Lawrence D.A., Andersen N., Davidova A., Beran O., Maresova V., Chalupa P. (2013). Cytokines and chemokines as biomarkers of community-acquired bacterial infection. Mediat. Inflamm..

[43] Knaus W.A., Draper E.A., Wagner D.P., Zimmerman J.E. (1985). Apache II: A severity of disease classification system. Crit. Care Med..

[44] Lim W.S., van der Eerden M.M., Laing R., Boersma W.G., Karalus N., Town G.I., Lewis S.A., Macfarlane J.T. (2003). Defining community acquired pneumonia severity on presentation to hospital: An international derivation and validation study. Thorax.

